# Toothbrush abrasivity in a long-term simulation on human dentin depends on brushing mode and bristle arrangement

**DOI:** 10.1371/journal.pone.0172060

**Published:** 2017-02-21

**Authors:** Mozhgan Bizhang, Ilka Schmidt, Yong-Hee Patricia Chun, Wolfgang H. Arnold, Stefan Zimmer

**Affiliations:** 1 Faculty of Health, School of Dentistry, Department of Operative and Preventive Dentistry, Witten/Herdecke University, Witten, Germany; 2 Department of Periodontics, Department of Cellular Systems and Anatomy, University of Texas Health Science Center at San Antonio, San Antonio, Texas, United States of America; 3 Department of Biological and Material Sciences in Dentistry, Witten/Herdecke University, Witten, Germany; University of Brescia, ITALY

## Abstract

**Objective:**

The aim of this study was to evaluate the susceptibility of dentin to brushing abrasion using four different toothbrushes (rotating-oscillating, sonic and two types of manual toothbrushes) with the same brushing forces.

**Methods:**

Dentin samples (n = 72) were selected from 72 impacted third molars. Half of the surface of dentin samples was covered with an adhesive tape, creating a protected and a freely exposed area in the same specimen. Brushing was performed with either a: sonic (Sonicare PowerUp, Philips GmbH, Hamburg, Germany), b: oscillating-rotating (Oral B Vitality Precisions Clean, Procter & Gamble, Schwalbach am Taunus, Germany) or two different manual toothbrushes c: flat trim brush head toothbrush (Dr. Best: Original, Glaxo-Smith-Kline, Bühl, Germany) and d: rippled-shaped brush head toothbrush (Blend-a-Dent, Complete V-Interdental, Blend-a-med, Schwalbach, Germany) in a custom made automatic brushing machine. The brushing force was set to 2 N and a whitening toothpaste (RDA = 150) was used. The simulation period was performed over a calculated period to mimic a brushing behavior of two times a day brushing for eight years and six months. Dentin loss was quantitatively determined by profilometry and statistically analyzed by Wilcoxon and Mann-Whitney-U Test (p < 0.05).

**Results:**

The mean (standard deviation) surface loss was 21.03 (±1.26) μm for the sonic toothbrush, 15.71 (±0.85) μm for the oscillating-rotating toothbrush, 6.13 (±1.24) μm for the manual toothbrush with flat trim brush head and 2.50 (±0.43) μm for the manual toothbrush with rippled-shaped brush head. Differences between all groups were statistically significant at p<0.05.

**Conclusion:**

Using the same brushing force and a highly abrasive toothpaste, manual toothbrushes are significantly less abrasive compared to power toothbrushes for an 8.5—year simulation.

## Introduction

The increasing elderly population in many developed countries is expected to retain their teeth into old age [1]. Simultaneously, the number of exposed root surfaces and non-carious cervical lesions in elderly people is steadily increasing[2]. The need for adequate prevention and treatment of this condition is of high relevance. The treatment options for non-carious cervical lesion include either to watch and wait or to intervene early with restorations [3–5]. Clinically, the most critical step is the detection of the disease and the identification of the cause before the etiological factors can be addressed. Non-carious cervical lesions often are the result of substance loss that results from mechanical interaction between toothbrush, toothpaste and tooth. Frictional forces are increased by small particles contained in toothpaste [6, 7]. The abrasivity is modified by the type of toothbrush and the applied brushing force [8].

Today power toothbrushes are widely used and power toothbrushes show more benefits with regard to reducing gingivitis and plaque in comparison to manual toothbrushes in short- and long-term observation periods [9, 10]. However, it is plausible that the use of power toothbrushes—albeit more effective for plaque removal—might be associated with a higher risk of loss of tooth substance. According to a recent review article, a comparison of power and manual brushes revealed that power brushes are less abrasive than, or similarly abrasive as manual brushes. Additionally, the comparison of different power toothbrushes has shown significant differences in abrasivity [11]. A recent consensus report concluded that there is currently no evidence from studies regarding the development or progression of non-carious cervical lesions [12].

Non-carious cervical lesions are seen daily by clinicians in dental practice. It is unknown whether the higher cleaning efficacy of power toothbrushes with an abrasive toothpaste used over long period of time might be harmful for hard tissues. Thus, the aim of this study was to evaluate the brushing abrasion of dentin using four different toothbrushes with toothpaste with a relative dentin abrasiveness index (RDA) of 150 in a simulated long-term setting. Two power toothbrushes and two manual toothbrushes were included. The null hypothesis was that there are no differences between the tested toothbrushes in abrasiveness.

## Materials and methods

### Specimen collection

Based on an effect size of 1.0, a power of 80% and a significance level of 5% (*p* < 0.05), the sample size was determined to be 18 per group, resulting in a total of 72 specimens. The sample size calculation was performed with the G*Power software (version 3.0;University of Duesseldorf) [13]. The protocol for the collection of teeth for this *in vitro* study was approved by the ethics committee of Witten/Herdecke University, Witten, Germany (No. 116/2013). A questionnaire asked patients of the dental clinic of Witten/Herdecke University on their first visit whether they “allow use of the extracted teeth for research”. Only extracted teeth with prior written consent were collected. In addition, all patients were verbally informed that their extracted molars would be used for research purposes. Extracted teeth were de-identified before they were passed on to the investigator.

### Specimen preparation

Seventy-two extracted human molars were used to generate dentin specimens. Molars were inspected for imperfections in the surface. Teeth with cracks, caries, discolorations or loss of hard tissue were excluded. Teeth were stored in 0.7% NaCl solution containing 0.1% thymol. Cylindrical dentin specimens (6 mm in diameter and 2 mm high) were prepared using a trephine bur (Hager & Meisinger GmbH, Neuss, Germany). Only one specimen was prepared from each tooth. The enamel layer was removed with a diamond bur until the dentin layer was exposed, which was verified under a light microscope (10x magnification). After removal of the enamel, the dentin surface was flattened and progressively polished with abrasive paper (up to 1000 grit) using a polishing machine (EXAKT, Norderstedt, Germany).

### Toothbrushing machine

**Fig 1:**
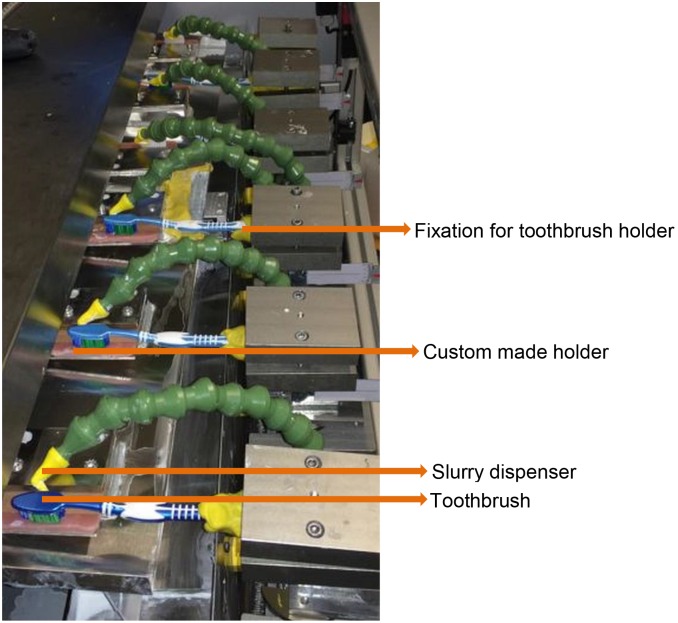
DentTest, Department of Operative and Preventive Dentistry, Witten/Herdecke University and Ingpuls GmbH Bochum, German.

The toothbrushing station (DentTest, Department of Operative and Preventive Dentistry, Witten/Herdecke University and Ingpuls GmbH Bochum, Germany) was developed for the simulation of the tooth cleaning process using both power and manual toothbrushes. The tooth brushing machine included six holders for toothbrushes (Fig 1). Each toothbrush worked on up to three specimens. The holders for the toothbrushes were customized for the toothbrush handle with silicone putty (Eurosil, Henry Schein, Melville, NY, USA) to hold the toothbrush in place. The specimens were mounted with standardized key lock fixations. The bristles of the toothbrush were aligned without pressure contacting the specimen surface in perpendicular fashion.

A linear cleaning movement of 3 cm length was selected for the experiments with power and manual toothbrushes. The movement length was sufficient to cover the specimens’ surfaces. A force of 2 N was chosen for brushing. The cleaning force was generated using a compressing spring and an extending screw.

Before the brushing procedure was initiated, half of the dentin surface was covered with an adhesive tape (Tesa, Beiersdorf, Hamburg, Germany) parallel to the long axis of the direction of the brushing movement [14].

### Toothbrushes and slurry

**Fig 2:**
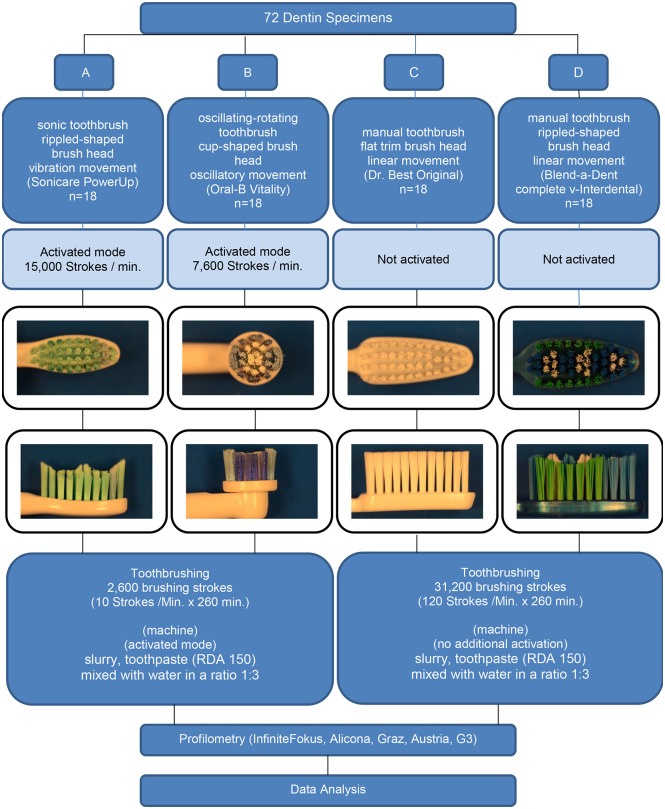
Study design.

Experiments were performed using two power and two manual toothbrushes (Fig 2).

- Group A: Sonic toothbrush (Sonicare PowerUp, Philips GmbH, Hamburg, Germany)
- Group B: Oscillating-rotating toothbrush (Oral B Vitality Precisions Clean, Procter & Gamble; Schwalbach am Taunus, Germany)
- Group C: Manual toothbrush, flat trim brush head (Dr. Best Original, Glaxo-Smith-Kline, Bühl, Germany),
- Group D: Manual toothbrush, rippled-shaped brush head (Blend-a-Dent, Complete V-Interdental, Blend-a-med, Schwalbach am Taunus, Germany)

To prepare a slurry, a toothpaste (Dentalux, Dental Kosmetik GmbH, Dresden, Germany) was mixed with water in a ratio of 1:3 according to the EN ISO 11609:2010 standard (Dentistry-Toothpastes: Requirements, test methods and marking). According to the manufacturer’s information, the RDA value of the toothpaste was 150. The ingredients of the toothpaste included water, sorbitol, hydrated silica, potassium citrate, propylene glycol, glycerin, sodium bicarbonate, sodium C 14–16 olefin sulfonate, cellulose, gum, aroma, tetrapotassium pyrophosphate, sodium fluoride, sodium, saccharin, allantoin, sodium methylparaben, titanium dioxide, limonene, and CI 74160 (1450 ppm fluoride).

### Brushing experiment

Specimens were randomly allocated to four groups (Fig 2). 18 specimens were assigned to each toothbrush. The total brushing strokes were calculated to be equivalent to 8.5 years of brushing, based on a brushing time of 120 seconds twice-daily of all teeth [15]. Based on this estimation, the maximum contact time for one tooth surface per day is 5 seconds [16]. The total brushing time was calculated to be 260 min. The brush head should be replaced after 45 days (a typical time period to replace the brush). This represents 270 minutes of cumulative use for 28 teeth (72 surfaces) with 5 s brushing per day. The total surface of the three specimens with 6 mm diameter approximately equalled the surface of one tooth. A brushing time of 5 s per day for 8.5 years is equivalent to 260 min. Therefore, the brushing time of 260 min was selected for the study. The movement of the power toothbrushes differs from brushing with a manual toothbrush. The manufacturer’s manual indicates that “toothbrushes based on a sonic technology has bristles that move side-to-side”. With oscillating-rotating technology, the brush head oscillates from a center point but does not rotate in a full circle [17]. Considering these differences in brushing movement, each sample was submitted to 31,200 brushing strokes at a rate of 120 strokes per minute for manual toothbrushes [18] and 2,600 brushing strokes at a rate of 10 strokes per minute for activated power toothbrushes. Brushing movements were executed with the slurry applied to the surface of the specimens. The flow rate of the slurry was set at 10 ml/minute. Specimens were rinsed with tap water for 30 seconds and received new slurry automatically every 2 minutes. The cleaning force was set to 2 N, and the cleaning movement was set at 3 cm longitudinally. After the final cleaning run all samples were stored in saline to avoid sample disintegration due to dehydration.

### Measurement of dentin loss

**Fig 3:**
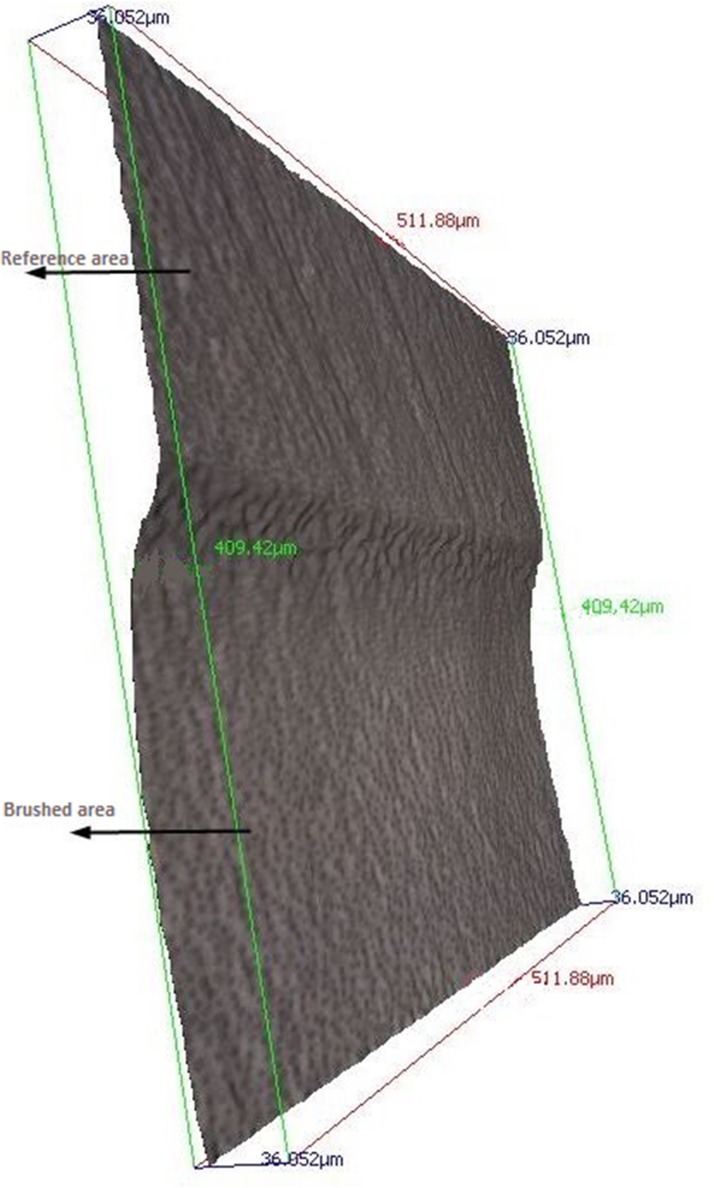
3D image of profilometry. Frontal view of a scan with the reference area on top and the brushed area on the bottom.

After removal of the adhesive tape, the dentin specimen was carefully dried with cotton rolls and briefly air dried to retain moisture in the specimen. Differences in surface abrasion between the exposed and protected area of the dentin specimens were evaluated using optical profilometry (InfiniteFokus G3, Alicona, Graz, Austria) with the corresponding software (IFM 2.2). For quantitative measurements of the surface abrasion, a 3D image at 20 x magnification and resolution was taken at the border of the exposed and protected dentin surface to include both areas in equal parts. The scanned area was equally divided between the area covered with tape and the area exposed to toothbrushing (Fig 3).

**Fig 4:**
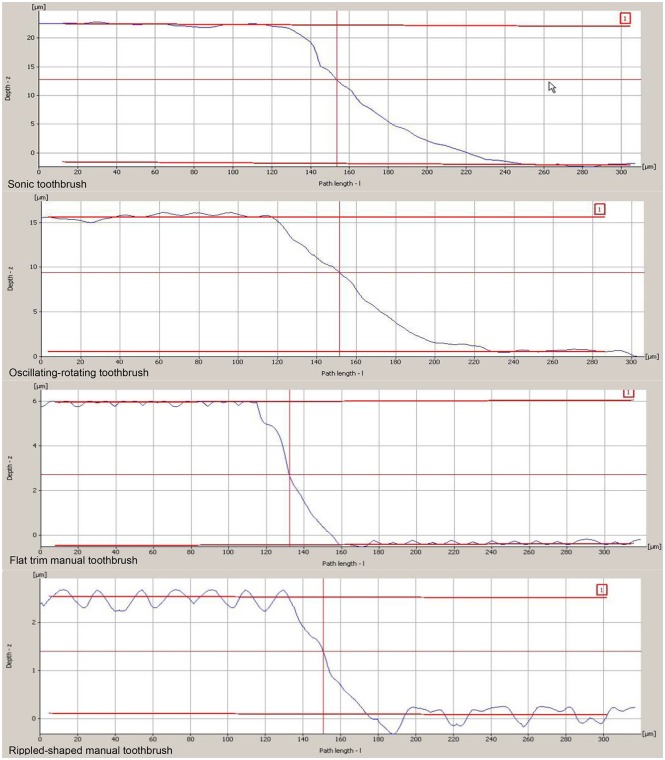
Measurement of dentin loss using profilometry for different toothbrushes for different toothbrushes. (A) Sonic toothbrush, (B) Oscillating-rotating toothbrush, (C) Flat trim manual toothbrush (D) Rippled-shaped manual toothbrush.

Measurements were made under moist conditions at all times. Before each measurement, the sample’s surface was covered with distilled water for 30 seconds. Excess of water was blotted with absorbent tissue without touching the specimen surface. Surface scans were performed with a 20x magnification lens and a vertical depth of 150 nm. 15 scans were taken for each specimen, starting in the center of the specimen, followed by scans in 50 μm steps above and below the starting point. Fifteen parallel lines with a length of 300 μm and a distance of 50 μm to each other were drawn within this selected field to determine the relative surface height after brushing. The mean of 15 measurements of the surface height served as the primary outcome of this study. Immediately after the completion of the profilometry measurement, specimens were placed back into saline. (Fig 4A–4D)

### Data analysis

IBM SPSS Statistics 20 was used for statistical analysis. For each toothbrush, mean differences between brushed and reference areas were calculated with standard deviation and 95% confidence intervals. The Kolmogorov-Smirnov test showed a homogeneous distribution of the data. A one-way ANOVA with post hoc Bonferroni corrections was performed for further statistical analysis. The significance was set at p<0.05. Alpha was adjusted to avoid alpha-error accumulation with regard to multiple paired comparisons. Therefore, p<0.01 was calculated for the primary outcome.

## Results and discussion

### Results

**Fig 5:**
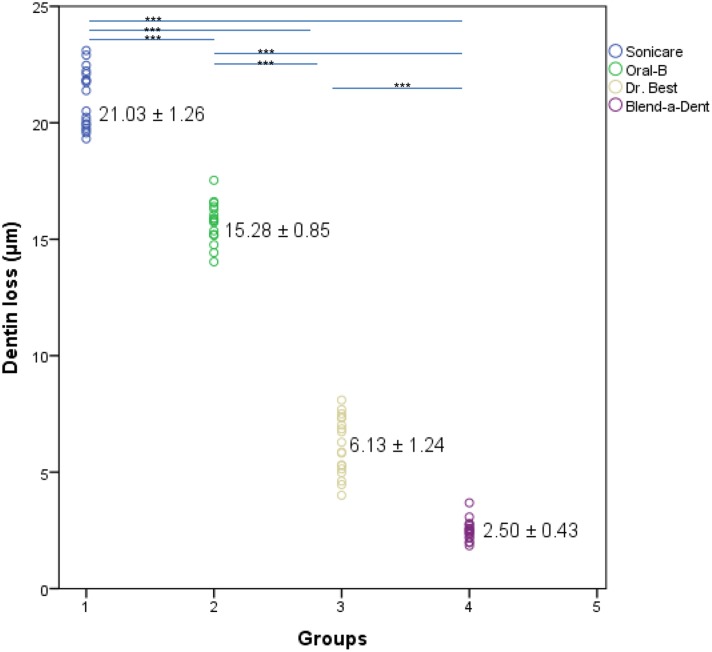
Scatterplot of the relationship between dentin loss and toothbrushes. Mean and standard deviation change of dentin loss (μm) after brushing with different toothbrushes and different strokes (***p<0.001). Horizontal bars indicate statistically significant differences between groups for power and manual toothbrushes, *** p < 0.001, one-way ANOVA with post hoc.

The power toothbrushes caused significantly higher dentin abrasion compared to the manual toothbrushes using the same brushing force and time. The mean (± standard deviation, and 95% confidence level 95%CI) surface loss was 21.03 (±1.26, 95%CI = 20.41–21.66) μm for the sonic toothbrush, 15.71 (±0.85, 95%CI = 15.28–16.13) μm for the oscillating-rotating toothbrush, 6.13 (±1.24, 95%CI = 5.51–6.75) μm for the flat trim manual toothbrush, and 2.50 (±0.43, 95%CI = 2.28–2.71) μm for the rippled-shaped manual toothbrush. Highest dentin abrasion was measured for sonic toothbrush and lowest for the rippled-shaped manual toothbrush. The order of the highest to the lowest abrasion after simulation of eight years and six months is as follows: sonic toothbrush, oscillating-rotating toothbrush, manual toothbrush with flat trim, rippled-shaped manual toothbrush. The dentin loss was significantly different between all groups (p< 0.001) (Table 1, Fig 5, data in supporting information file S1 Table.

**Table 1:** Descriptive data of dentin loss (μm).

Design of toothbrush	Toothbrush	Manufacturer	Stroke number	Mean	Standard deviation	95% confidence interval
**• Sonic**	Sonicare PowerUp	Philips GmbH	2610	21.03	1.26	20.41–21.66
**• Oscillating-rotating**	Oral B Vitality Precisions Clean	Procter & Gamble	2610	15.28	0.85	15.28–16.13
**• ManualFlat-trim**	Dr. Best Original	Glaxo-Smith-Kline	31200	6.13	1.24	5.51–6.75
**• ManualRipple-shaped**	Blend-a-Dent Complete V-Interdental	Blend-a-med	31200	2.50	0.43	2.28–2.71

## Discussion

This study evaluated the effect of four toothbrushes on dentin abrasion over a simulated long-term period *in vitro*. Patients with cervical abrasions were found to have less plaque than those without abrasions [19]. *In vitro* studies showed that the abrasive effect of tooth brushing on the gingiva depends on the direction and frequency of the brushing movement and other factors such as applied force as well as quality and arrangement of the toothbrush bristles [20]. Our study shows that abrasion of dentin by tooth brushing depends to a significant degree on the type of toothbrush. Two power toothbrushes with two different modalities (side-to-side-action, oscillating-rotating) and two different manual toothbrushes were tested in the present study. Hence, the null hypothesis of this study was rejected.

Brushing force is a crucial factor for the development of abrasions. A brushing force of 3 N in an *in-vitro* study was shown to produce increased loss of dentin *in vitro* [21]. Loss of enamel can be induced when challenged with acid under a similar brushing force of 2.5 N [22]. Brushing forces from 0.9 N [23], 1.7 N [24], 2.5 N [25], 3 N [26], or 4 N [27] were reported in users of power toothbrushes. For manual toothbrushes, brushing forces ranged from 1.6 to 3.23 N [23, 28–31],. In *in vitro* settings with a toothbrushing machine a brushing force of 5 N is used to generate toothbrushing abrasions for manual toothbrushes [32]. Furthermore, the variability of the mean recorded force for power toothbrushes ranges from 0.8 N and 2.7 N [33] suggesting that each toothbrush model has a specific profile for brushing force. When the brushing force of subjects with multiple recessions was measured, they were found to use 3.75 N which was 1.63 N higher than in subjects without recession [34]. If the brushing force is standardized at 2 N for manual and power toothbrushes, no differences in abrasion of dentin were found [35]. Interestingly, brushing without toothpaste resulted in minimal abrasion in dentin [35]. A manual brushing force of 250 g (equal to 2.5 N) with a manual toothbrush (Oral-B) and 150 g (equal to 1.5 N) with a power toothbrush (Braun Oral-B Excel) did not result in differences in lost dentin [36]. When the brushing load was altered for power (0.9 N) and for manual (2.5 N) toothbrushes at a constant stroke number, the abrasion of dentin decreased in the power toothbrush group [37]. Therefore, abrasions generated by power toothbrushes are not contributed by the brushing force. Since the goal of our study was to examine the effect of the type of toothbrush and bristle arrangement, the brushing force was standardized for both manual and power brushes. A moderate brushing force of 2 N was selected to determine the influence of mode of action and bristle configuration of the toothbrushes on dentin abrasion.

The abrasions of dental hard tissue increase with the rate of brushing movements [38]. The frequency of brushing movements is higher in manual vs. activated power toothbrushes. When the same brushing force was used for power and manual toothbrushes *in vitro* the power toothbrush operated with 374 strokes compared to a manual toothbrush with 1,500 strokes [39]. According to our observations, manual toothbrushing requires more than 12 times more strokes than using a power toothbrush. The stroke number used in this study was adopted from *in vitro* studies using 120 strokes/min for manual [40–42] and 10 strokes/min for power toothbrushes [43]. Our results showed that a high stroke frequency with manual toothbrushes was less abrasive than the power toothbrushes with low stroke frequency at the same brushing force. Activated sonic toothbrushes have an average oscillation rate of 15,000 strokes/min; activated oscillating-rotating toothbrushes execute 7,600 strokes/min. The activated mode renders it unnecessary for the user to move the head in order to execute the “brushing” motion as executed for manual brushing. Instead, the user positions the vibrating brush head to the teeth with minimal movement. According to the physical definition of work, work results from a constant force of magnitude F on a point that moves a displacement (s) in the direction of the force W = F x s. If the force is constant and the distance will be increased, the work (abrasion) will be higher. Using the same brushing force in the present study, the bristles of the power toothbrushes traveled a longer distance in the sonic toothbrush compared to the oscillating-rotating toothbrush or the manual toothbrushes. The greater movement distance of the bristles of the sonic toothbrush may have contributed to the greater loss of dentin.

Besides the brushing movement, the tested toothbrushes differed in the design of the brush heads. The design of the brush head and the arrangement of the bristles may influence the abrasivity of the toothpaste. In our study, all toothbrushes had parallel bristles arranged in tufts, but differed in tuft number, configuration of tufts and tuft length (flat form vs. rippled-shaped). Among the power toothbrushes the rippled-shaped form toothbrush produced more abrasions compared to the flat trim brush. In contrast, among the manual toothbrush, the rippled-shaped toothbrush produced less abrasion when compared to the flat trim manual brush. Among manual toothbrushes the flat form toothbrush had more filaments contacting the dentin surfaces than the rippled-shaped, possibly transporting toothpaste across the dentin surface more efficiently. Since the bristles made only light contact to the dentin surface and no additional vertical force was applied, the shorter bristles of the rippled-shaped manual toothbrush may not have contacted the dentin surface. The oscillating-rotating toothbrush has a cup-shaped brush head, which is smaller than the sonic toothbrush with a brush head similar to a manual toothbrush. Furthermore, the movement frequency of the oscillating-rotating toothbrush is lower than in the sonic toothbrush. For the Oral-B power toothbrush, all flat trim tufts were in contact with the dentin through their oscillating-rotating movements. For the sonic power toothbrush, the wiping movement may apply shear forces to the dentin surface when longer bristles execute wiping movements upon activation. Two studies found that manual and power toothbrush appear to differ in the transportation of toothpaste and the resulting abrasion of sound dentin specimens [36, 39]. Their results showed higher dentin loss by manual compared to power toothbrushes. The first study examined the effects of an oscillating-rotating toothbrush (Oral-B 3D) with 52 strokes/surface (1 stroke/min for the duration of 52 min) and of a manual toothbrush with 12,500 strokes/surface (240 strokes/min for 52 min). Dentin loss was found to be greater for the manual toothbrush than for sonic or oscillating-rotating toothbrushes [36]. The second study with the oscillating-rotating toothbrush (Oral-B D9) was performed on an empty shaker bath which produced a controlled brush movement of 44 strokes/min (374 strokes total) in comparison to the ADA toothbrush used by the brushing machine for a total of 1,500 strokes [39].

*In vitro* studies for the examination of dental abrasions offer a standardized setting that eliminates patient related parameters such as different cleaning force and time as well as environmental factors. To determine the abrasivity of different toothbrushes, *in vitro* studies allow the comparison of different types and designs of toothbrushes under controlled conditions [6, 22, 44].

Slurry was generated according to EN ISO 11609:2010 standard (Dentistry-Toothpastes: Requirements, test methods and marking). Water and toothpaste with RDA 150 were mixed in a ratio of 1:3 before application to the specimens. Toothbrushes *per se* do not cause significant differences in abrasion of softened human enamel [45]. However, increasing the abrasivity of the toothpaste caused an increase in surface loss of enamel samples [45]. In conclusion, RDA values of toothpastes seem to have a greater impact on dentin loss than the hardness of bristles [46]. No significant damage to dental tissues is detected when teeth are brushed with water [47]. The toothpaste used in this study with RDA 150 is considered highly abrasive, as is typical of whitening toothpastes. The RDA scale ranges from 0 to 250. A toothpaste with a higher RDA was selected in view of the increasing popularity of whitening toothpastes.

Horizontal brushing movements were executed in the toothbrushing machine to simulate the “scrub technique” as the most widespread brushing technique among children and adults [48, 49]. In the patient setting, the individual brushing load and strokes of subjects is very difficult to reconstruct.

## Conclusion

This long-term *in vitro* study showed that the formation of dentin abrasions depends on the brushing mode, and bristle arrangement for manual and power toothbrushes used with a toothpaste with high abrasivity. Abrasions of dentin were higher in power toothbrushes compared to manual toothbrushes. The highest dentin loss was observed using a sonic power toothbrush and the lowest using the rippled-shaped manual toothbrush.

## Supporting information

S1 Table Data_final_english. (XLSX)
